# Assessment of drug harm by final-year medical students and ChatGPT – a comparative analysis

**DOI:** 10.1186/s13011-026-00762-1

**Published:** 2026-09-12

**Authors:** Isak Näslund, Michael Ott, Alexander Vucins, Ursula Werneke

**Affiliations:** 1 Department of Clinical Sciences, Psychiatry, Sunderby Hospital - Norrbotten, Umeå University, Umeå, Sweden; 2 Department of Public Health and Clinical Medicine, Umeå University, Umeå, Sweden; 3 Department of Psychiatry, Sunderby Hospital, Luleå, Sweden

**Keywords:** ChatGPT, Medical students, Recreational drugs, Substance use disorders, Artificial intelligence, Illicit drugs, Alcohol

## Abstract

**Background:**

Artificial intelligence (AI) is becoming increasingly integrated into medical education and clinical practice. Junior medical staff often act as first-line responders in substance-related presentations, yet their perceptions of drug-related harm are not well characterised. It is unclear how their assessments compare with harm ratings generated by emerging AI tools. This study aimed to compare final-year medical students’ harm ratings of recreational drugs and alcohol to users and society with those generated by ChatGPT.

**Methods:**

110 final-year medical students (59.1% response rate) rated the harmfulness of 28 recreational drugs to individual users on a 1–10 scale and ranked seven drug classes for societal harm. ChatGPT-4 (paid subscription version) generated 40 response sets in five conversations through repeated administration of the same survey. Discrepancies in harm ratings to users and rankings of societal harm were analysed descriptively.

**Results:**

Students rated their drug harm knowledge at 5.8/10 (SD 1.7) and their education on drug harm at 5.3/10 (SD 1.8). ChatGPT rated its drug harm knowledge at 8.3/10 (SD 1.2). The mean harm-to-user scores across the 28 substances were 6.7 (SD 1.8) for students and 6.4 (SD 0.8) across the repeated ChatGPT response sets. Both groups identified heroin, crack cocaine, and fentanyl as the most harmful substances. Differences were observed for less harmful substances: students rated snus (smokeless tobacco), laughing gas, and cannabis least harmful, whereas ChatGPT rated magic mushrooms, DMT/ayahuasca, and LSD least harmful. ChatGPT ratings were more similar to those of previously published expert panels.

**Conclusions:**

Students and ChatGPT strongly agreed on the most harmful substances but differed in their assessments of moderately and minimally harmful drugs. These differences suggest that perceptions of drug harm are shaped not only by pharmacology and toxicity but also by social and cultural context, local patterns of use, clinical exposure and experience, and medical training. AI may be of limited value when the evidence base is sparse, contested, or difficult to trace. The lower confidence among students suggests that further research is needed to better understand educational needs in drug-harm assessment and the critical appraisal of AI-generated evidence.

**Supplementary Information:**

The online version contains supplementary material available at https://doi.org/10.1186/s13011-026-00762-1.

## Background

Recreational drugs and alcohol continue to pose major global public health challenges. According to the United Nations Office on Drugs and Crime, recreational drug use has increased by more than 20% over the past decade, with an estimated 292 million people having used drugs in 2022 [1], and over 1,000 new psychoactive substances identified since 2013 [2]. However, evaluating and comparing the harms associated with different drugs remains complex. Harm varies by substance type, route of administration, dose, frequency and duration of use, and by the physical, psychological, and social consequences experienced. Perceptions of harm also differ across cultural or geographical contexts, further complicating assessment.

Drug harm ratings are important for informing both public health policy and clinical decision-making. Expert panels in the UK, EU, Australia, and New Zealand [3–7] have evaluated the relative harm of drugs to individuals and society. In the absence of objective comparative measures of overall drug harm, these expert consensus assessments represent the best available benchmark for evaluating the relative harms of psychoactive substances. Across all four regions, alcohol has consistently been ranked as among the most harmful substances overall. Yet, it remains unclear how these aggregate harm rankings translate to the clinical level, where decisions must be tailored to individual patients. Moreover, expert consensus may not reflect the perspectives of junior medical staff, who frequently act as first-line responders in situations involving acute intoxication or withdrawal. Little is known about how final-year medical students who are about to enter clinical practice evaluate drug and alcohol harm, or how well they feel prepared for this task.

Advances in artificial intelligence (AI), particularly large language models (LLMs) like ChatGPT-4 (OpenAI) and subsequent versions are expected to influence medical education and clinical practice [8–9]. Already in 2023, a study had shown that an earlier version of ChatGPT (ChatGPT-3) performed at or near the passing level on all three exams of the United States Medical Licensing Examination (USMLE) [10]. Although this finding related specifically to the USMLE and should not be interpreted as proven competence across all medical licensing systems, it highlighted the potential of LLMs to perform at a level comparable to trainees on certain knowledge-based assessments. Promising diagnostic abilities, including image-based tasks, had even been predicted earlier [11]. It is plausible that AI systems may soon support clinical decision-making in mental health care, enhancing precision, treatment efficacy, and personalised care through its ability to recognise patterns and integrate large multimodal datasets [12]. At the same time, its capabilities may remain limited by many unanswered clinical, scientific, and ethical questions [13]. However, responsible integration of AI into clinical practice requires critical evaluation of its capabilities and limitations, including in contexts such as drug harm assessment.

The role of AI in medical education is rapidly evolving. A survey among 390 medical students in the US conducted in 2022 found that students recognised the importance of AI in medicine but felt that opportunities to learn about AI-related topics were limited. Fundamental AI concepts, appropriate applications and advantages and limitations of AI were identified as important areas for inclusion in medical training [14]. A recent review of AI literacy among healthcare professionals and students in the Americas concluded that the adoption of AI in medical education remains geographically variable and highlighted the importance of strengthening AI literacy in both educational and clinical settings [15]. In view of these developments, it is important to better understand how future clinicians evaluate drug-related harm and how their assessments compare with those generated by emerging.

AI-based tools. Therefore, this study aimed to examine how final-year medical students rate the harm of a range of recreational drugs and alcohol to both individual users and society and compare these assessments with harm ratings generated by ChatGPT-4.

## Methods

### Study design

We conducted a cross-sectional survey to assess perceptions of the harmfulness of recreational drugs to the individual user and to society as a whole. The societal perspective was included because it formed part of the methodology used by the expert panels [3–7] and because the perceived harms of a substance may differ depending on whether they are evaluated from an individual or societal perspective. The survey was administered in September–October 2023 to all final-year medical students at Umeå University, Sweden. Drug harm ratings were also obtained from ChatGPT-4, which was the most recent publicly available version of the model at the time of data collection.

### Ethics approval and consent to participate

Under the Swedish Ethics Review Act (2003:460), this study did not require formal ethical approval because it did not involve the collection of sensitive personal data but only professional opinions and basic non-sensitive personal information (age and sex). Nevertheless, we obtained an advisory opinion (rådgivande yttrande) from the Swedish National Ethical Review Authority, which raised no concerns (DNR 2023-02057-01). The study was conducted in accordance with the ethical principles of the Declaration of Helsinki.

Participants were informed in the electronic invitation letter accompanying the survey link about the purpose and procedures of the study. The invitation explained that participation was voluntary and that the survey was anonymous. Completion and submission of the survey implied informed consent. No compensation was provided for participation.

### Sample

We invited all final-year medical students at Umeå University (10th and 11th academic half-year of the then 11-half-year undergraduate medical programme) during the autumn term of 2023 to participate. Hereafter, we refer to this group simply as students. These students were selected because, under Swedish regulations, they are entitled to work under supervision in clinical settings and are therefore likely to be first responders in substance-related medical emergencies. Participants confirmed their voluntary participation at the start of the survey.

Data from ChatGPT were collected by pasting the survey text (Appendix) into the chat interface, ensuring that identical instructions and questions were provided to both the AI and students. Four researchers, each using separate OpenAI accounts, generated a total of 40 AI-generated response sets in five ChatGPT conversations. Three conversations generated ten response sets each, one generated six response sets, and one generated four response sets. The fifth conversation became necessary because two conversations did not generate the target of ten response sets. For all conversations, a web browser was used as the interface (Microsoft Edge for two conversations and Google Chrome for three). No regeneration or follow-up prompting was used. All researchers used a paid subscription version of ChatGPT-4 (Appendix). Repeated response sets were not treated as independent ratings analogous to the medical students’ responses. Rather, they were used to characterise the distribution of outputs generated by a single LLM under repeated administration of the questionnaire.

### Survey procedures

A student administrator distributed the survey via email to all eligible students, with reminders sent at two and four weeks. Additionally, the survey link was shared in two closed Facebook groups used exclusively by the student cohort.

The survey (Appendix) was created in Microsoft Forms and administered in Swedish, as the undergraduate medical programme is taught in Swedish and all study participants could be expected to be fluent in the language. The survey began with a brief explanation of the term *harm to users* and clarified that the questions concerning prescription drugs referred to non-medical use. Participants were then asked to rate the harm of 28 recreational substances on this basis and were instructed to skip any substances they did not know rather than guess.

The list of substances (Table 1) was compiled in consultation with psychiatrists specialising in substance use disorder, representatives from local law enforcement, scientific literature, and media reports to ensure that the substances reflected those commonly encountered in clinical practice in the region. In cases where substances were considered interchangeable in the Swedish context, they were grouped into a single item, for example, amphetamine and methamphetamine (meth-/amphetamine). Familiar drug names or abbreviations were used throughout.

**Table 1:** Substances included in the study, grouped by substance category

Substance categories	Individual drugs/ substance sub-categories	Comments
Anabolic steroids	Not further specified	
Alcohol	Not further specified	
Cannabinoids	Cannabis	
	Spice	Synthetic cannabis
Central nervous system (CNS) depressants	Benzodiazepines	
	Butane gas	
	Pregabalin	
	Zopiclone, Zolpidem	Referred to as Z-drugs in the analysis
	GHB	Gamma-hydroxybutyrate
Central stimulants	Amphetamine/	In Sweden, amphetamine and methamphetamine are often used interchangeably. Rated as meth-/amphetamine.
	Methamphetamine	
	Cocaine	
	Crack cocaine	
	Methylphenidate	
Opioids usually taken orally	Buprenorphine	
	Methadone	
	Oxycodone	
	Tramadol	
Opioids commonly injected	Fentanyl	
	Heroin	
Psychedelics, hallucinogens, and other dissociative drugs	Bath salts	PV-8, MDPV, 3-mmc/4-methylmetcathione (MMC)/Mephedrone
		Dimethyltryptamine
	DMT/Ayahuasca	3,4-methylenedioxymethamphetamine
	Ecstasy/MDMA	Nitrous oxide
	Ketamine	Lysergic acid diethylamide
	Laughing gas	Referred to as magic mushrooms in the analysis
	LSD	
	Magic mushrooms/ Psilocybin	
Tobacco products	Cigarettes	
	Snus	Swedish tobacco product consisting of a pouch of tobacco leaves to be placed under the lip for absorption by the oral mucosa

For the final section of the survey, the concept of *harm to society* was defined both in the Swedish and in the global context, after which participants were asked to rank seven broader drug categories in terms of their societal harm.

### Outcomes

Two primary outcomes were assessed: harm to the individual user (harm to user) and perceived harm to society (harm to society). Harm to society was further divided into two subcategories, covering the global and Swedish perspectives. To ensure consistency with previous harm assessment research [3–5], we used established conceptual definitions.

*Harm to user* was defined as the negative effects a substance may have on an individual user, including mortality, morbidity, addiction, impaired mental ability, risk of life disruption, involvement in crime, loss of property and relationships, and reduced ability to maintain self-sufficiency. Participants were asked to *rate* the harm to users for the 28 identified substances on a continuous 1–10 scale, where 1 indicated completely harmless and 10 indicated extremely harmful.

*Harm to society* was defined as the broader societal impact of a substance, considering the prevalence of use and associated negative consequences, such as psychological and physical harm to others, crime, environmental damage, family difficulties, economic cost, and damage to community cohesion. Participants were asked to *rank* seven categories in terms of societal harm: alcohol, benzodiazepines, cannabis, injectable opioids, prescription opioids, psychedelics, and stimulants. Tobacco products (cigarettes and snus) and anabolic steroids were excluded because they rarely cause acute intoxication or withdrawal and are primarily associated with long-term harm profiles.

### Statistical analysis

Descriptive statistics were used to summarise the harm ratings. For each substance, we calculated the proportion of non-responses among students. Mean harm-to-user scores were calculated for each substance separately for students and ChatGPT. Comparisons between students and ChatGPT were based on a sample of students and repeated outputs from an LLM. The repeated outputs were not treated as independent observations. Differences in mean harm scores between students and ChatGPT were examined descriptively for both individual substances and across all substances. Standard deviations for the ChatGPT response sets were presented solely as descriptive measures of variation across the observed repeated outputs and are not interpreted as estimates based on an independent sample.

For the societal harm rankings, responses were visualised using a heat map, where a rank of 1 (darkest shading) indicated the highest perceived societal harm, and a rank of 7 (lightest shading) indicated the lowest perceived harm.

To assess potential non-response bias, we compared the sex distribution of the survey respondents with that of non-responders based on the full cohort of final-year medical students during the autumn term of 2023 using a chi-square test. Missing responses were treated as indicators of unfamiliarity with a substance, as specified in the survey instructions.

The statistical analysis was conducted with IBM SPSS Statistics (Version 29.0.1.0). All visualisations were performed using Microsoft Excel (Version 16.0.17230.42306). Summary data used for the figures, means, and standard deviations are provided in the appendix.

## Results

### Survey turnout

Of 186 students, 110 (59.1%) completed the survey. In total, 122 questionnaires were submitted, of which twelve were excluded because the respondents indicated that they were not in their final year. Among the 110 participants, 40 (36.4%) were male, 69 (62.7%) female, and one (0.9%) preferred not to disclose sex. Fifty-one (46.4%) were ≤ 25 years, 58 (52.7%) > 25 years, and one (0.9%) did not disclose age. For comparison, among all eligible students, 75 (40.3%) were male and 111 (59.7%) female. The sex distribution did not differ significantly between responders and non-responders (*p* = 0.236).

### Non-response rates for harm-to-user ratings

**Fig. 1:**
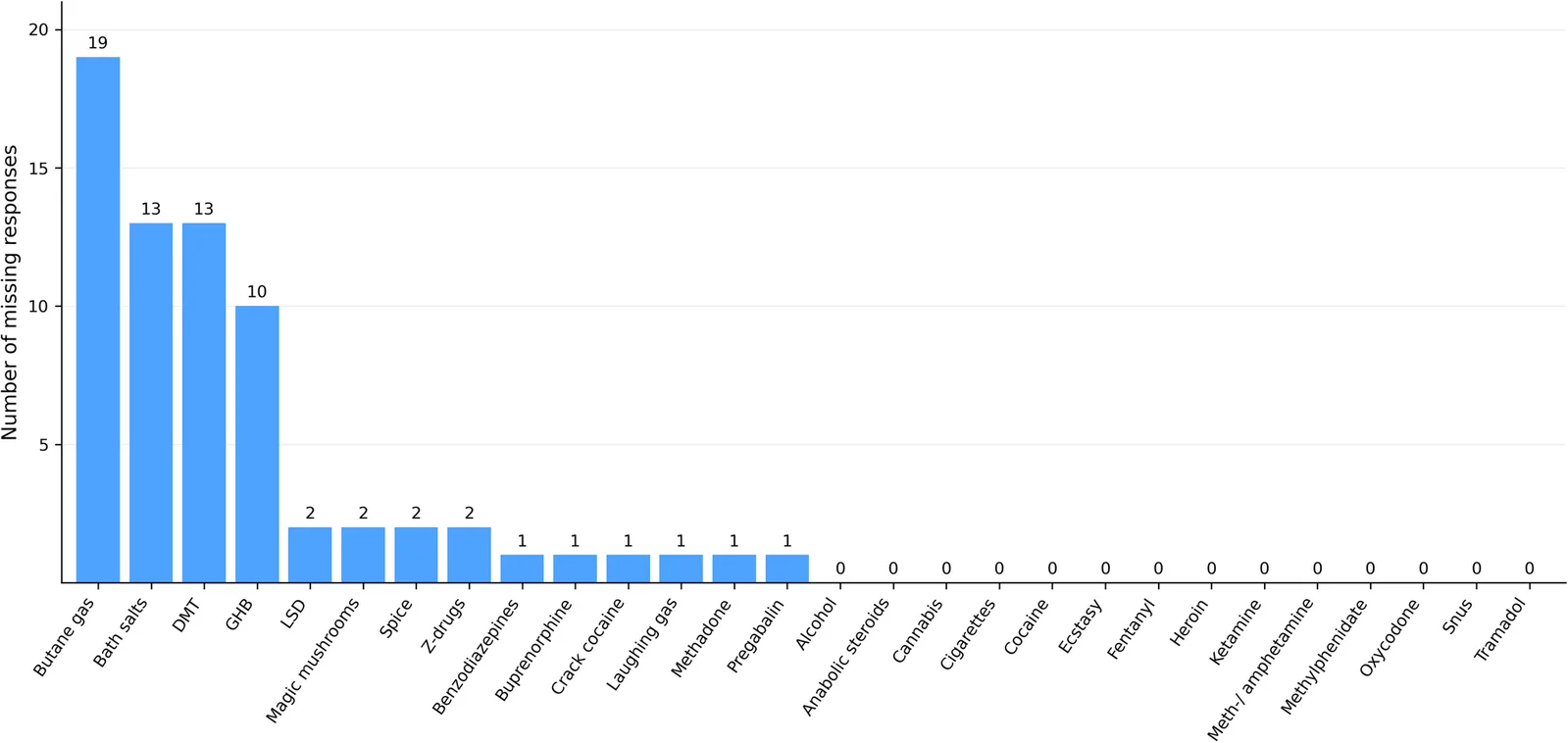
Proportion of missing responses of students for 28 individual substances (*n* = 110). Numbers above the bars indicate the number of students who did not provide a rating for that substance. For explanation of substance names and abbreviations see Table 1.

Responses were complete for 14 of 28 substances (50.0%). For ten substances (35.7%), one or two responses were missing. For the remaining four substances (14.3%), more than two responses were missing. The substances with the highest proportion of missing ratings were butane gas (17.2%), DMT (11.8%), bath salts (11.8%), and GHB (9.1%) (Fig. 1).

Students rated their overall knowledge of drug-related harm at a mean of 5.8/10 (SD 1.7) and the extent of their education on drug harm at 5.3/10 (SD 1.8). Across the repeated ChatGPT response sets, ChatGPT rated its knowledge of drug harm as 8.3/10 (SD 1.2) when prompted to use the same scale.

### Harm-to-user ratings

The mean harm-to-user score across all substances was 6.7 (SD 1.8) for students. Across the repeated ChatGPT response sets, the mean was 6.4 (SD 0.8). Both students and ChatGPT rated heroin, crack cocaine, and fentanyl as the most harmful substances.

Ratings diverged thereafter. Students rated cocaine as the fourth most harmful substance and meth-/amphetamine as fifth. In contrast, ChatGPT ranked bath salts fourth and alcohol equal with meth-/amphetamine as the fifth most harmful.

**Fig. 2:**
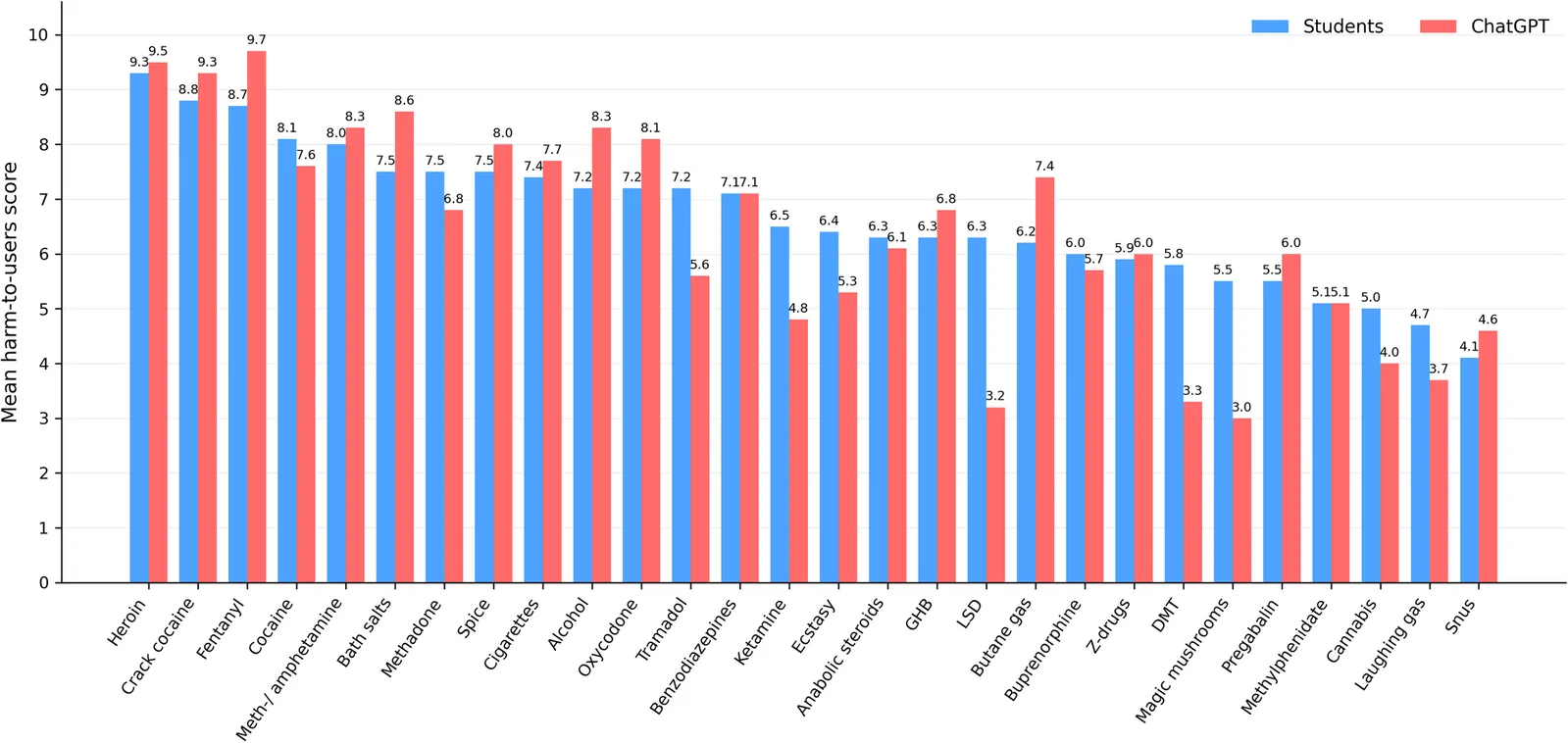
Harm-to-user ratings by students and ChatGPT across 28 substances. Ratings range from 1 (completely harmless) to 10 (extremely harmful). Substances are ordered by the mean harm-to-user ratings provided by the students (blue) highest to lowest. ChatGPT ratings are shown for comparison (red). Numbers above the bars present the mean harm-to-user score. The highest ratings were assigned to heroin by students and to fentanyl by ChatGPT, whereas the lowest ratings were assigned to snus by students and magic mushrooms by ChatGPT.

For the lowest harm ratings, students identified snus, laughing gas, and cannabis as the least harmful substances. ChatGPT identified magic mushrooms, DMT/ayahuasca, and LSD as the least harmful (Fig. 2; see Appendix for full rankings).

### Comparison of mean harm-to-user scores

Students assigned higher mean harm-to-user scores than ChatGPT for LSD, magic mushrooms, DMT, ketamine, tramadol, ecstasy, cannabis, laughing gas, methadone, cocaine, buprenorphine, and anabolic steroids. In contrast, ChatGPT assigned higher mean harm-to-user scores than students for butane gas, bath salts, alcohol, fentanyl, oxycodone, pregabalin, GHB, snus, spice, crack cocaine, cigarettes, meth-/amphetamine, heroin, and Z-drugs. Benzodiazepines and methylphenidate received the same mean harm-to-user scores from students and ChatGPT.

**Fig. 3:**
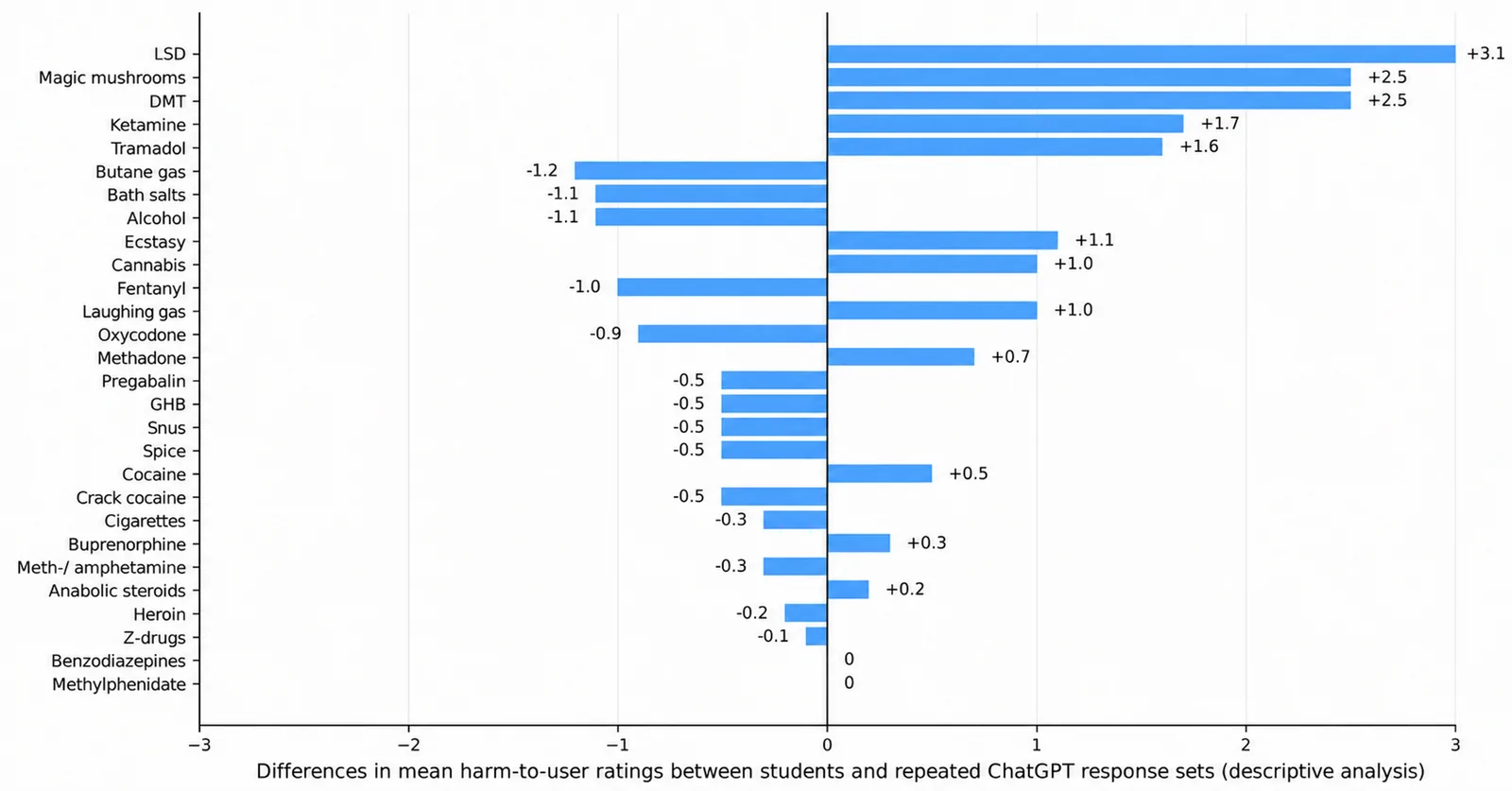
Differences in mean harm-to-user ratings between students and ChatGPT across 28 substances. Bars show the difference in mean harm-to-user ratings (student rating minus ChatGPT rating) A value of 0 indicates no difference. Positive values indicate higher mean harm-to-user ratings by students; negative values indicate higher mean harm-to-user ratings by ChatGPT

### Harm-to-society rankings

Students and ChatGPT ranked drug classes according to their perceived harm to society from both a Swedish and a global perspective. In both perspectives, alcohol was ranked as the most harmful, and psychedelics and cannabis as the least and second least harmful, respectively.

**Fig. 4:**
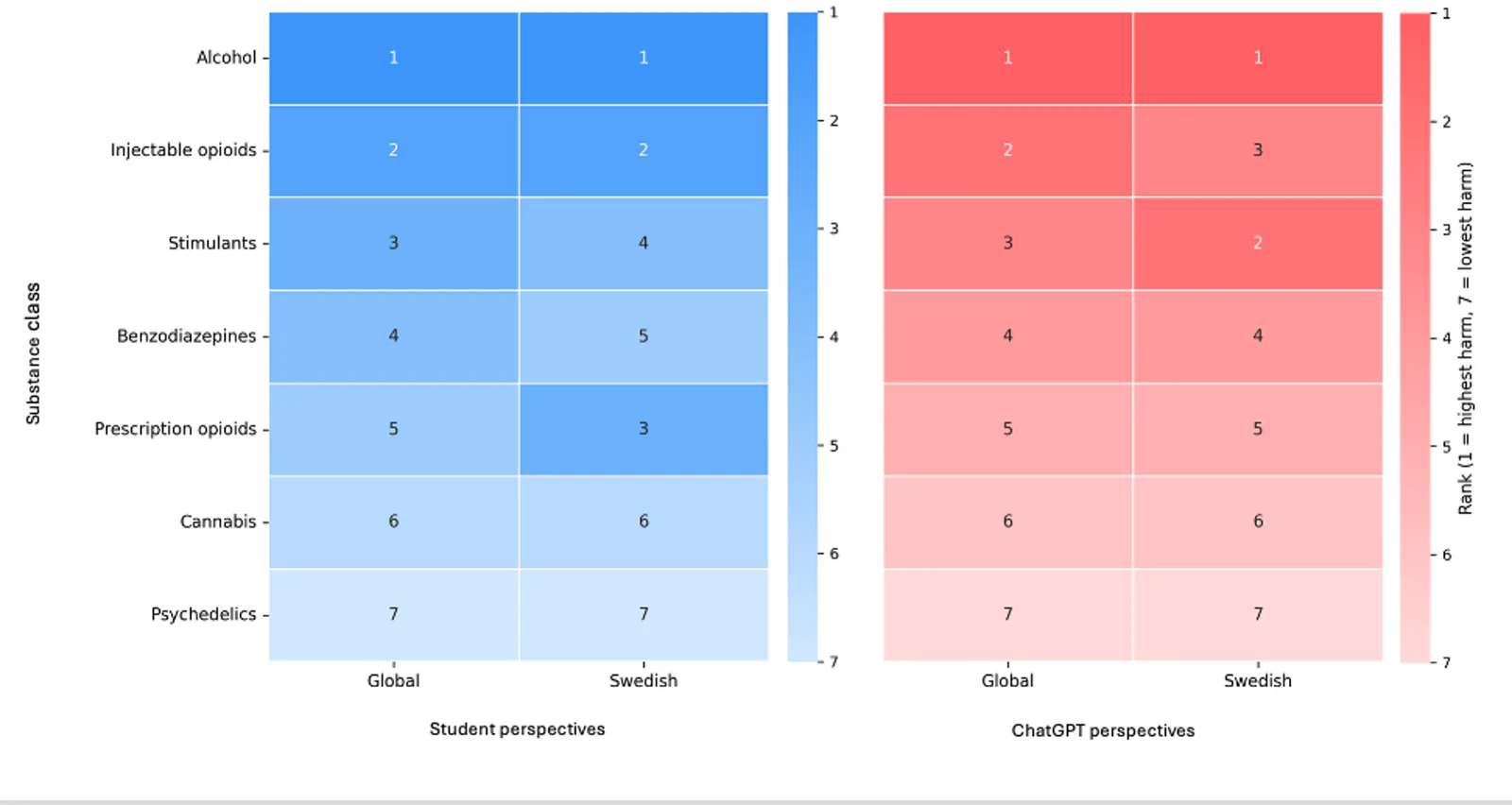
Rankings of harm-to-society associated with drug classes by students and Chat GPT. Rankings are shown for the global and Swedish societal perspectives (1 = most harmful, 7 = least harmful). Darker shading indicates higher perceived harm

There was complete agreement between students and ChatGPT in the global harm-to-society rankings. In contrast, rankings differed in the Swedish societal context. Students ranked the societal harm of injectable and prescription opioids higher, and the harm of stimulants and benzodiazepines lower compared with ChatGPT (Fig. 4).

## Discussion

### Findings

Our findings show that students and ChatGPT broadly agreed on the substances posing the highest harm to the individual user, namely heroin, crack cocaine, and fentanyl. Differences were observed for substances for which harm perceptions are more strongly influenced by cultural framing and clinical experience. Students rated psychedelics and cannabis as more harmful than ChatGPT, whereas ChatGPT rated alcohol and several synthetic or high-potency substances (for example, bath salts and spice) as more harmful than students. For harm to society, students and ChatGPT showed complete agreement in the global context, but their rankings diverged in the Swedish context. Notably, students also rated both their knowledge of drug harm and the extent of their medical education in this area as limited.

### Comparison with other studies

We identified five previous studies that ranked drug harm, using expert panels and comparable assessment frameworks [3–7] (Table 2). These studies were conducted in the UK [3, 4], the European Union (EU) [5], Australia [6], and New Zealand [7]. One senior author was involved in all five, which likely contributed to the consistency observed across them. Overall, there was substantial agreement, particularly across the four most recent studies [4–7]. However, some variation was evident, which may reflect changes in drug markets over time, especially the emergence of synthetic psychoactive substances [16, 17].

Heroin was ranked among the three most harmful substances in four studies [3–6]. In the earliest study from 2007, cocaine was also ranked among the three most harmful [3], whereas later studies placed greater emphasis on other stimulants, namely crack cocaine [4, 5] and methamphetamine [6, 7]. In the most recent study from 2023, synthetic cannabinoids were ranked among the three most harmful, while non-prescription opioids were only ranked fifth [7].

Across the four most recent studies, alcohol was consistently rated as the most harmful substance overall and as the most harmful to society [4–7]. For harm to the individual user, alcohol was ranked fourth in all four studies. Conversely, hallucinogens, including LSD and magic mushrooms, were consistently ranked among the three least harmful substances [4–7].

**Table 2:** Previous expert panels studies ranking drug harm

Study	Nutt et al., 2007 [3]	Nutt et al., 2010 [4]	Amsterdam et al., 2015 [5]	Bonomo et al., 2019 [6]	Crossin et al., 2023 [7]
Location	UK	UK	EU	Australia	New Zealand
N harm categories	9	16	16	16	17
N participants	8-29^b^ experts	15^c^ experts	40 experts	25 experts	23 experts
N substances ranked	20	20	20	22	23
3 substances ranked as overall most harmful^a^	Heroin, Cocaine Barbiturates	Alcohol, Heroin, Crack cocaine	Alcohol, Heroin, Crack cocaine	Alcohol, Crystal meth Heroin	Alcohol, Methamphetamine Synthetic cannabinoids
3 substances ranked as overall least harmful^a^	Ecstasy, Alkylnitrates, Khat	LSD, Buprenorphine, Mushrooms	LSD, Buprenorphine Magic mushrooms	LSD/ magic mushrooms ENDS, Kava^d^	Hallucinogens Kava, Nitrous oxide
Rank^a^ alcohol					
Harm overall	5	1	1	1	1
Harm to user	–	4	4	4	4
Harm to society	–	1	1	1	1
Rank^a^ synthetic cannabis					
	Not included	Not included	Not included		
Harm overall				9	3
Harm to user				8	1
Harm to society				11	5
Rank^a^ cannabis					
Harm overall	11	8	8	13	6
Harm to user	–	12	7	15	9
Harm to society	–	5	10	6	3
Rank^a^ magic mushrooms, psilocybin				LSD & mushrooms	Hallucinogens
	Not included				
Harm overall		20	20	20	21
Harm to user		19	20	19	22
Harm to society		20	20	20	16

^a^Rankings are presented in descending order from most to least harmful, derived or estimated from the figures in the published articles

^b^The study involved two expert groups: 29 addiction psychiatrists and a multidisciplinary panel of 8–16 experts

^c^Information not provided in the article; source Wikipedia https://en.wikipedia.org/wiki/Drug_Science

^d^Piper methysticum

DMT : Dimethyltryptamine, ENDS: electronic nicotine delivery systems, EU: European Union, LSD: Lysergic acid diethylamide, N: number, N/A not applicable as harm to users and harm to society determined through different methods, UK: United Kingdom

In another study from Germany, two separate questionnaire-based surveys were conducted in which 101 and 36 experts in addiction medicine ranked the harmfulness of 30 substances [18]. Crack cocaine, methamphetamine, and heroin were considered the most harmful, followed by alcohol ranked fourth and cocaine fifth. Synthetic cannabinoids ranked ninth and cannabis 16th. Prescription opioids were rated among the least harmful substances.

In the same project, two additional surveys were conducted among drug users (*n* = 100 and *n* = 44). For the 13 substances with at least 20 responses, harm rankings were calculated [19]. Similar to the expert evaluations, heroin, cocaine, and amphetamine were rated as the most harmful overall. Synthetic cannabinoids were ranked fourth and alcohol fifth. Cannabis, buprenorphine, and psychotropic mushrooms (i.e., mushrooms containing psilocybin) were ranked among the least harmful.

With respect to harm to the individual user, students and ChatGPT broadly agreed with previous expert panel evaluations: injectable or high-potency opioids and potent stimulants were consistently rated as highly harmful, while psychedelics and cannabis were placed among the least harmful substances. ChatGPT showed close agreement with expert rankings for both harm to users and harm to society. In contrast, students rated the harm of LSD and psilocybin-containing mushrooms to the individual higher than both the expert panels and ChatGPT, although they agreed with the low rankings of harm to society. Students’ higher ratings of psychedelics may reflect prevailing legal restrictions and cultural attitudes toward these substances in Sweden [20]. Alternatively, ChatGPT’s lower ratings may reflect its reliance on aggregated global evidence derived from expert assessments, including those presented here, rather than contextual factors specific to Sweden. However, the similarity between ChatGPT’s ratings and expert panels should not be interpreted as evidence that ChatGPT can replace expert panels, expert judgement, or scientific evidence. Nor should this finding be taken to imply greater validity of AI-generated responses.

In line with previous expert-panel studies using comparable methods [3–7], both students and ChatGPT ranked alcohol as the most harmful substance to society. For harm to the individual user, ChatGPT showed close agreement with the expert panels and ranked alcohol as the fifth most harmful substance. In contrast, students ranked alcohol as the tenth most harmful substance to the individual user, considerably lower than both ChatGPT and the expert evaluations. Interestingly, the students’ rankings were more consistent with harm ratings reported in the German surveys among experts and drug users [18, 19].

The reasons for these differences are not entirely clear. Cultural norms surrounding alcohol use may influence perceptions of harm, particularly in countries where alcohol consumption is common and socially accepted. Additionally, the harmful effects of alcohol often accumulate gradually and may be more apparent with greater clinical experience. Harm perceptions among students may therefore evolve over time as their exposure to alcohol-related morbidity increases.

In accordance with the ratings reported by drug users in the German surveys [18], final-year students and ChatGPT assigned lower harm ratings to cannabis than the expert panels, which had placed cannabis in the mid-range [4–7, 18] (Table 2). The reasons for this difference are not fully clear. One possible explanation is a lower perceived risk among young medical practitioners, a pattern also observed in the younger Swedish population more broadly [21]. Additionally, the harm of cannabis is not uniform and depends on the product used. Synthetic cannabinoids, for example, carry substantially higher risks than plant-based cannabis preparations [22]. This pattern was also evident in the New Zealand expert-panel study, in which synthetic cannabinoids received high harm ratings [7]. In our study, cannabis and spice (a synthetic cannabinoid product) were rated separately and spice was indeed rated substantially more harmful to the individual than cannabis. Furthermore, as with alcohol, many of the harmful effects of cannabis may accumulate over time and depend on frequency and duration of use [23]. These factors may contribute to lower perceived harm among students who have had limited exposure to long-term cannabis-related morbidity. Given the heterogeneity of perceptions of drug-related harm and the limited comparative empirical evidence available, expert panels provide the most appropriate benchmark against which student and ChatGPT ratings can be compared.

### Limitations

An inherent feature of studies assessing perceived drug harm is that rankings and ratings are subjective. However, this limitation applies equally to the expert-panel studies on which previous drug harm frameworks are based. There is also a potential for selection bias, as the views of responders may differ from those of non-responders; this cannot be fully eliminated in survey research. The response rate in our study was 59%, close to the commonly recommended benchmark of approximately 60% [24] and within the range of the average response rate reported for online surveys (44%–67%) [25, 26]. In addition, the sex distribution did not differ significantly between responders and non-responders, suggesting that sex-related non-response bias was low. However, other sources of selection bias cannot be excluded.

The current study was conducted in one single institution with a substance list tailored to regional clinical relevance. The findings may not be generalisable to students from other regions in Sweden or students from other countries, where perceptions of drug-related harm may be influenced by cultural norms, legal frameworks, patterns of substance use, and availability of specific substances in a different way. We have already discussed this in the context of alcohol, which occupies a distinctive cultural position in many societies. Similar considerations apply to snus. In Sweden, both traditional tobacco-containing snus (brown snus) and nicotine pouches (white snus) are legally available and widely used, having a long-standing cultural acceptance. In contrast, the sale of snus in either form is prohibited in most other EU countries, and the regulations regarding the consumption of nicotine pouches vary considerably. Consequently, Swedish students may perceive snus as less harmful than students from countries where its use is uncommon, heavily restricted, or socially stigmatised. More broadly, perceptions of drug-related harm are likely to be shaped by cultural and regulatory contexts, which should be considered when interpreting and generalising findings.

A further limitation is that multiple ChatGPT response sets were generated within the same conversation and remained visible throughout the session. Therefore, contextual influences between rating rounds cannot be excluded, and the repeated response sets cannot be regarded as independent observations. This clustering within conversations may also have reduced the observed variability among the ChatGPT response sets. Future studies intending to perform inferential comparisons should generate each response set in a separate independent conversation.

More generally, the processes by which LLMs generate responses are not fully transparent, underscoring the need for further research on how AI-generated outputs should be interpreted and applied in clinical and educational contexts. Finally, although students were specifically asked to rate their own knowledge, and using ChatGPT would have required additional effort, we cannot completely exclude the possibility that some may have used ChatGPT when completing the survey.

The repeated ChatGPT response sets should not be interpreted as equivalent to responses from 40 independent human participants. Whereas variation among students reflects differences between individuals, variation among ChatGPT response sets reflects the model giving somewhat different answers when asked the same question repeatedly. Consequently, for the ChatGPT response sets, the reported means and standard deviations describe the variation across repeated model outputs rather than the characteristics of a population of AI respondents. There may also be differences in response sets depending on whether paid or unpaid versions are used. While all four researchers in this study used paid subscriptions, students may be more likely to use free AI models. For example, a survey conducted at a US college between December 2024 and February 2025 found that 89.3% of AI users reported using the free version of ChatGPT [27].

Another limitation is the exclusion of tobacco products from the societal harm rankings despite their well-established contribution to morbidity, mortality, and public health burden. Tobacco products were excluded because the study focused on substances commonly associated with intoxication, acute withdrawal, and emergency or psychiatric presentations. In addition, combining cigarettes, snus, and other nicotine products into a single category would have been problematic, as their patterns of use and associated harms differ substantially. The exclusion of tobacco should therefore not be interpreted as implying that tobacco products are associated with limited harm but rather reflects the specific clinical focus and design of the study. Furthermore, tobacco products do not typically induce acute confusional states, psychosis, or aggression, which were important considerations in the context of this study.

## Conclusion

Students and ChatGPT agreed on which substances were most harmful to individual users, but differences were observed for substances in the mid- and low-harm range. For these substances, ChatGPT’s ratings showed closer agreement with those of previous expert panels, which currently represent the most appropriate benchmark against which student and ChatGPT ratings can be compared.

These differences suggest that perceptions of drug harm are shaped not only by pharmacology and toxicity but also by social and cultural context, local patterns of use, clinical exposure and experience, and medical training. In contrast, LLMs such as ChatGPT draw on harm perceptions derived from global information patterns, which may not fully capture factors that are more evident to clinicians in local settings. Therefore, AI-generated assessments should not be automatically or directly translated into clinical decision-making for individual patients; they can support –but not replace– clinical judgement.

AI may be of limited value when the evidence base is sparse, contested, or difficult to trace. The lower confidence reported by students suggests that further research is required to better understand educational needs in drug-harm assessment and the critical appraisal of evidence generated by artificial intelligence.

## Supplementary Information

Below is the link to the electronic supplementary material.

Supplementary Material 1

## Data Availability

All data underlying the figures are provided in the appendix. The original dataset can be made available upon reasonable request.

## Abbreviations

AI: Artificial intelligence
Meth-/ amphetamine: Amphetamine and methamphetamine
CNS: Central nervous system
DMT: Dimethyltryptamine
GHB: Gamma-hydroxybutyrate
LLM: Large language models
LSD: Lysergic acid diethylamide
MMC: Methylmetcathione
MDMA: 3,4-methylenedioxymethamphetamine
MDPV: Methylenedioxypyrovalerone
PV-8: α-Pyrrolidinoheptaphenone
SD: Standard deviation
UK: United Kingdom
USMLE: United States Medical License Examination
