# Supplementary material for: Assessment of drug harm by final-year medical students and ChatGPT – a comparative analysis
Source: Subst Abuse Treat Prev Policy. 2026 Sep 12;21:57. doi: 10.1186/s13011-026-00762-1 (PMC13587526; doi:10.1186/s13011-026-00762-1)
Supplement: Supplementary file 1 [file 13011_2026_762_MOESM1_ESM.docx]

**APPENDIX**

**Questionnaire as submitted to final year medical students via Microsoft Forms and to ChatGPT-4**

Information regarding anonymity was provided in the invitation letter that accompanied the survey link.

Translated from the original Swedish version.

| In this survey, you are asked to rate various recreational drugs from most harmful to least harmful to the individual and society. Before you begin please note the following:  *Harm to the individual*, refers to negative effects on the user, such as mortality, morbidity, addiction, impaired mental ability, risk of life disruption, involvement in crime, loss of property and relationships, and reduced ability to maintain self-sufficiency.  For questions concerning prescription medications, we refer to no-medical use (misuse) and not to medically prescribed use.  Please try to rate all substances, However if there is a substance you do not recognise or have no opinion about you may skip that item, We are seeking only you professional judgment. | |
| --- | --- |
|  | Response alternatives |
| Which term of the medical program are you in? | Term 11  Term 10  None of the above |
| Sex | Male  Female  Prefer not to say |
| Age | <25  25 or older  Prefer not to say |
| How would you rate your knowledge about the harmful effects of different drugs? | 1–10 with 1 = very poor and 10 = very good |
| How extensive has your education been regarding the harmful effects of different drugs? | 1–10 with 1 = very poor and 10 = very good |
| How harmful is [insert substance] for the individual?  Benzodiazepines   - Zopiclone/Zolpidem - Pregabalin (Lyrica) - Cannabis - Spice - Alcohol - Cigarettes - Snuff (Swedish snus) - Buprenorphine - Fentanyl - Heroin - Methadone - Oxycodone - Tramadol - Amphetamine/Methamphetamine - Bath salts (PV-8, MDPV, 3-mmc, 4-mmc, mephedrone) - Crack cocaine - Cocaine - Methylphenidate (Concerta, Ritalin) - Ecstasy (MDMA) - Ketamine - LSD - Magic mushroom/psilocybin - DMT / Ayahuasca - GHB - Anabolic steroids - Laughing gas - Butane gas | 1–10 with 1 = not harmful at all and 10 = extremely harmful |
| Rank the follwong groups of drugs from the most to the least harmful to society in Sweden. Consider the prevalence of the drug in relation to its negative effects on society. Negative effect on society are defined as psychological and physical harm to others, crime, environmental damage, family difficulties, economic cost, and damage to community cohesion. Hover over the items and use the arrows to rank them. | - Alcohol - Benzodiazepines and similar drugs - Central stimulants such as amphetamine and cocaine - Cannabis - Non-injectable opiates such as tablets or fentanyl patches - Injectable opiates such as heroin - Psychedelics such as LSD and magic mushrooms |
| Rank the follwong groups of drugs from the most to the least harmful to society globally. Consider the prevalence of the drug in relation to its negative effects on society. Negative effect on society are defined as psychological and physical harm to others, crime, environmental damage, family difficulties, economic cost, and damage to community cohesion. Hover over the items and use the arrows to rank them. | - Alcohol - Benzodiazepines and similar drugs - Central stimulants such as amphetamine and cocaine - Cannabis - Non-injectable opiates such as tablets or fentanyl patches - Injectable opiates such as heroin - Psychedelics such as LSD and magic mushrooms |
| Notes:   1. “Narkotikapereparat (Swedish legal term) translated here as “recreational drugs” to reflect non-medical use context. 2. “Opiater” in the original survey is used in the broad clinical sense equivalent to “opioids”. | |

**Method for obtaining 40 ChatGPT ratings**

|  | **Rater 1** | **Rater 2** | **Rater 3** | **Rater 4** |
| --- | --- | --- | --- | --- |
| N ratings | 6  4 incomplete rounds | 2x 10 | 4  6 incomplete rounds | 10 |
| Chat session structure | One chat session | 2 chat sessions, 10 per round | One chat session | One chat session |
| Same prompt used in all rating rounds | Yes | Yes | Yes | Yes |
| Follow-up prompting/ regeneration allowed | No | No | No | No |
| ChatGPT version | 4 | 4 | 4 | 4 |
| Paid subscription | Yes | Yes | Yes | Yes |
| Interface (web browser or app) | Web browser interface | Web browser interface | Web browser interface | Web browser interface |
| Browser | Edge | First chat session Google Chrome,  second chat session Edge | Google Chrome | Google Chrome |

*Ten initial rating rounds did not yield numerical ratings and were therefore excluded. Ten additional rating rounds were subsequently completed, resulting in a total of 40 valid Chat GPT rounds.

**Background data for figures**

**Mean harm-to-users ratings (scores) for each substance according students and ChatGPT**

|  | **Students** | |  |  | **Chat-GPT**  **(n= 40 queries)** |  | **Final-year medical students vs. Chat GPT-4** |
| --- | --- | --- | --- | --- | --- | --- | --- |
| **Substance** | **N responses** | **Mean** | **SD** | **N**  **reponse sets** | **Mean** | **SD** | Difference of mean scores |
| Heroin | 110 | 9.3 | 0.9 | 40 | 9.5 | 0.5 | –0.2 |
| Crack cocaine | 109 | 8.8 | 1.1 | 40 | 9.3 | 0.5 | –0.5 |
| Fentanyl | 110 | 8.7 | 1.6 | 40 | 9.7 | 0.5 | –1.0 |
| Cocaine | 110 | 8.1 | 1.3 | 40 | 7.6 | 0.7 | +0.5 |
| Meth- / amphetamine | 110 | 8.0 | 1.6 | 40 | 8.3 | 0.6 | –0.3 |
| Bath salts | 97 | 7.5 | 1.8 | 40 | 8.6 | 0.5 | –1.1 |
| Methadone | 109 | 7.5 | 1.7 | 40 | 6.8 | 0.9 | +0.7 |
| Spice | 108 | 7.5 | 1.6 | 40 | 8.0 | 0.6 | –0.5 |
| Cigarettes | 110 | 7.4 | 2.1 | 40 | 7.7 | 0.9 | –0.3 |
| Alcohol | 110 | 7.2 | 1.7 | 40 | 8.3 | 0.5 | –1.1 |
| Oxycodone | 110 | 7.2 | 1.4 | 40 | 8.1 | 0.4 | –0.9 |
| Tramadol | 110 | 7.2 | 1.5 | 40 | 5.6 | 0.8 | +1.6 |
| Benzodiazepines | 109 | 7.1 | 1.5 | 40 | 7.1 | 0.5 | 0.0 |
| Ketamine | 110 | 6.5 | 1.9 | 40 | 4.8 | 1.1 | +1.7 |
| Ecstasy | 110 | 6.4 | 2.1 | 40 | 5.3 | 1.0 | +1.1 |
| Anabolic steroids | 110 | 6.3 | 1.9 | 40 | 6.1 | 1.0 | +0.2 |
| GHB | 100 | 6.3 | 1.9 | 40 | 6.8 | 0.9 | –0.5 |
| LSD | 108 | 6.3 | 2.3 | 40 | 3.2 | 1.1 | +3.1 |
| Butane gas | 91 | 6.2 | 2.1 | 40 | 7.4 | 1.0 | –1.2 |
| Buprenorphine | 109 | 6.0 | 1.9 | 40 | 5.7 | 0.8 | +0.3 |
| Z-drugs | 108 | 5.9 | 1.6 | 40 | 6.0 | 0.7 | -0.1 |
| DMT | 97 | 5.8 | 2.2 | 40 | 3.3 | 1.2 | +2.5 |
| Magic mushrooms | 108 | 5.5 | 2.3 | 40 | 3.0 | 1.0 | +2.5 |
| Pregabalin | 109 | 5.5 | 1.7 | 40 | 6.0 | 0.5 | –0.5 |
| Methylphenidate | 110 | 5.1 | 1.9 | 40 | 5.1 | 0.9 | 0.0 |
| Cannabis | 110 | 5.0 | 2.1 | 40 | 4.0 | 0.9 | +1.0 |
| Laughing gas | 109 | 4.7 | 2.1 | 40 | 3.7 | 0.5 | +1.0 |
| Snus | 110 | 4.1 | 1.9 | 40 | 4.6 | 1.0 | –0.5 |
| All substances average | 107.5 | 6.7 | 1.8 | 40 | 6.4 | 0.8 | +0.3 |

1–10 scale, with 1 = completely harmless and 10 = extremely harmful
